# Effect of Placement of a Supraglottic Airway Device vs Endotracheal Intubation on Return of Spontaneous Circulation in Adults With Out-of-Hospital Cardiac Arrest in Taipei, Taiwan: A Cluster Randomized Clinical Trial

**DOI:** 10.1001/jamanetworkopen.2021.48871

**Published:** 2022-02-18

**Authors:** An-Fu Lee, Yu-Chun Chien, Bin-Chou Lee, Wen-Shuo Yang, Yao-Cheng Wang, Hao-Yang Lin, Edward Pei-Chuan Huang, Kah-Meng Chong, Jen-Tang Sun, Matthew Huei-Ming, Ming-Ju Hsieh, Wen-Chu Chiang

**Affiliations:** 1 Department of Emergency Medicine, National Taiwan University Hospital, Taipei City, Taiwan; 2 Emergency Medical Services Division, National Fire Agency, Ministry of the Interior, Taiwan; 3 Department of Emergency Medicine, Taipei City Hospital, Zhongxiao Branch, Taipei City, Taiwan; 4 Emergency Medical Services Division, Taipei City Fire Department, Taipei City, Taiwan; 5 Department of Emergency Medicine, National Taiwan University Hospital, Hsin-Chu Branch, Hsin-Chu City, Taiwan; 6 Department of Emergency Medicine, Far Eastern Memorial Hospital, New Taipei City, Taiwan; 7 Department of Emergency Medicine, National Taiwan University Hospital, Yun-Lin Branch, Douliu City, Taiwan

## Abstract

**Importance:**

Prehospital advanced airway management with either initial endotracheal intubation (ETI) or initial supraglottic airway (SGA) insertion in patients with out-of-hospital cardiac arrest (OHCA) remains controversial.

**Objective:**

To compare the effectiveness of ETI and SGA in patients with nontraumatic OHCA.

**Design, Setting, and Participants:**

The Supraglottic Airway Device vs Endotracheal intubation (SAVE) trial was a multicenter cluster randomized clinical trial conducted in Taipei City, Taiwan. Individuals aged 20 years or older who experienced nontraumatic OHCA requiring advanced airway management and were treated by participating emergency medical service agencies were enrolled from November 11, 2016, to December 31, 2019. The final day of follow-up was February 19, 2020.

**Interventions:**

Four advanced life support ambulance teams were divided into 2 randomization clusters, with each cluster assigned to either ETI or SGA in a biweekly period.

**Main Outcomes and Measures:**

The primary outcome of the SAVE trial was sustained return of spontaneous circulation (ROSC) (≥2 hours) after resuscitation. Secondary outcomes included prehospital ROSC, survival to hospital discharge, and favorable neurologic outcome, defined as a cerebral performance category score less than or equal to 2. Prespecified subgroups and the association between time to advanced airways were explored. Per protocol and intention-to-treat analysis were performed.

**Results:**

A total of 936 patients (517 in the ETI group and 419 in the SGA group) were included in the primary analysis (median age, 77 [IQR, 62-85] years; 569 men [60.8%]). The first-attempt airway success rates were 77% with ETI (n = 413) and 83% with SGA (n = 360). Sustained ROSC was 26.9% (n = 139) in the ETI group vs 25.8% (n = 108) in the SGA group. The odds ratio of sustained ROSC was 1.02 (95% CI, 0.98-1.06) in the ETI group vs SGA group. The odds ratio of ETA vs SGA was 1.04 (95% CI, 1.02-1.07) for prehospital ROSC, 1.00 (95% CI, 0.94-1.06) for survival to hospital discharge, and 0.99 (95% CI, 0.94-1.03) for cerebral performance category scores less than or equal to 2.

**Conclusions and Relevance:**

In this randomized clinical trial, among patients with OHCA, initial airway management with ETI did not result in a favorable outcome of sustained ROSC compared with SGA device insertion.

**Trial Registration:**

ClinicalTrials.gov Identifier: NCT02967952

## Key Points

**Question:**

What is the effect of placement of a supraglottic airway device compared with endotracheal intubation among adults with out-of-hospital cardiac arrest?

**Findings:**

In this cluster randomized clinical trial, a total of 968 patients who experienced out-of-hospital cardiac arrest were enrolled. There was no significant difference between the 2 groups, in which the proportion of sustained return of spontaneous circulations was 26.9% in the endotracheal intubation group and 25.8% in the supraglottic airway group.

**Meaning:**

This clinical trial showed no difference in the rates of sustained return of spontaneous circulation in initial prehospital airway management with the placement of a supraglottic airway vs endotracheal intubation.

## Teaser

This cluster randomized clinical trial compares sustained return of spontaneous circulation following endotracheal intubation vs supraglottic airway device insertion among patients who experience out-of-hospital cardiac arrest.

## Introduction

Out-of-hospital cardiac arrest (OHCA) causes the death of hundreds of thousands of people yearly worldwide,^1^ including more than 10 000 people per year in Taiwan.^2^ Less than 5% of patients can survive until hospital discharge.^3^ Prehospital management includes basic and advanced life support, which are vital to patient outcomes.^4^ Tools in airway management are controversial in randomized clinical trials.^5,6^ Wang et al^6^ found that initial supraglottic airway (SGA) insertion was associated with greater 72-hour survival compared with initial endotracheal tube insertion; however, the first-attempt success rate was relatively low at 51% during the study period, compared with 69% in the study conducted by Benger et al.^5^ Benger et al stated that, in terms of 30-day favorable functional outcome, SGA devices are similar to endotracheal intubation (ETI).^5^

Intubation in patients with OHCA started in 2002 in Taipei, Taiwan. Paramedics were the earliest trained and authorized personnel to perform out-of-hospital intubation in metropolitan Asia.^7^ In a study conducted in Taipei, among patients with OHCA, 18% received ETI and 24% received SGA insertion; 58% of the remaining patients received bag-valve-mask ventilation.^8^ Quality control of airway management includes protocol-based single-attempt intubation and end-tidal capnographic data to prevent the need for esophageal intubation. The quality of chest compression is also monitored via automated external defibrillator records, in-ambulance videos, and randomly selected for scene supervision.

One retrospective cohort study conducted in Taipei revealed that successful ETI in patients following OHCA in the prehospital setting was associated with improved odds of sustained return of spontaneous circulation (ROSC) for 2 hours or more, survival to discharge, and favorable neurologic outcome compared with SGA insertion.^8^ Further high-quality data, such as randomized studies, are needed to strengthen the findings.

To determine the best approach for advanced airway management, we performed a cluster randomized clinical trial to compare the outcomes of either initial ETI or initial SGA insertion in adults following OHCA. We hypothesized that patients who experienced OHCA receiving ETI would have higher sustained ROSC rates than those receiving SGA.

## Methods

### Study Design and Setting

The Supraglottic Airway Device vs Endotracheal Intubation (SAVE) trial was a multicenter cluster randomized clinical trial. The trial protocol is available in Supplement 1. This study followed the Consolidated Standards of Reporting Trials (CONSORT) reporting guideline. The study protocol was approved by the institutional review board of the National Taiwan University Hospital, and eligible patients were enrolled automatically under the waiver of informed consent. The trial enrolled patients from November 11, 2016, to December 31, 2019, and the final day of follow-up was February 19, 2020. There are 4 advanced life support ambulance teams to serve the whole Taipei City. All paramedics in the advanced life support ambulance teams were recruited. According to the requirements of the Taiwan Ministry of Health and Welfare, each paramedic would have completed 1280 hours of training. They are trained and authorized to perform both ETI and SGA insertion and intravenous injections of medications.

### Patient Populations

The trial included patients with nontraumatic OHCA aged 20 years or older who were treated by the participating emergency medical service agencies and required advanced airway management. The exclusion criteria included (1) resuscitation deemed inappropriate (rigor mortis or livor mortis), (2) not suitable for ETI (ie, the inability to open the patient’s mouth wide enough for laryngoscope insertion), (3) not suitable for SGA (eg, preexisting tracheostomy), (4) cardiac arrest during transportation to the hospital, (5) family’s do-not-resuscitate request at the scene, (6) ROSC at the scene and no need for advanced airway support, and (7) airway devices (ETI or SGA) had been established before paramedics arrived.

### Randomization

The randomization was based on clusters of biweekly periods during the study from November 11, 2016, to December 31, 2019. Four advanced life support ambulance teams were divided into 2 randomization clusters. Each cluster was assigned to either ETI or SGA in a biweekly period. The study center would instruct the clusters to alternatively change to either the ETI or SGA insertion according to the sequence random allocation. A total of 164 randomization units were generated under this randomization process. The detailed randomization scheme is illustrated in the eFigure in Supplement 2. Per-protocol analysis was performed initially but the results of intention-to-treat analysis are also included. To guarantee a balanced per-protocol case number in the final analysis, we decided the allocation periods of ETI vs SGA in the fashion of 3 periods of ETI to 2 periods of SGA (3:2 ratio), which was based on the baseline of the first attempt success rates of ETI and SGA in the background data of the Taipei emergency medical service system (ie, the success rate of 60% with ETI vs 90% with SGA).

### Intervention

The intervention in our trial was either an initial SGA insertion or an initial ETI. The SGA was a second-generation SGA device (i-gel; Intersurgical Ltd). Tracheal intubation was performed using direct laryngoscopy but not video laryngoscopy during the study.

The protocol limited the insertion attempts of both SGA and ETI groups to once. End-tidal capnographic data to prevent esophageal intubation were also applied to the ETI group. If the first advanced airway attempt failed, rescue airway management, including ETI or SGA insertion or bag-valve-mask ventilation, was allowed. Apart from advanced airway management, all other care delivered at the scene followed the standard resuscitation guidelines.^9^ The paramedics participating in the trial did not receive additional training programs, and advanced airway management reflected the daily practice of their work.

### Outcomes

The primary outcome was sustained ROSC (defined as ROSC≥2 hours) after cardiac arrest, which differed from the primary outcome listed on the original trial registration. The primary outcome was changed to sustained ROSC before the trial began on November 11, 2016. Because the admission of patients with OHCA might be delayed in overcrowded emergency departments in Taiwan, we chose sustained ROSC as the primary outcome rather than survival to admission. In addition, the primary outcome with sustained ROSC was well validated and commonly reported in previous studies in Taiwan.^10,11^ The secondary outcomes included survival to hospital discharge and favorable neurological outcome (defined as cerebral performance category scores 1 or 2). For a comprehensive understanding of the effect of intubation on all survival status following OHCA, we analyzed prehospital ROSC as one of the secondary outcomes, which was not included in the original trial registration.

We also collected the Utstein-based registry of the patients with OHCA,^8,10,11^ which consisted of the following factors: patient demographic characteristics (age and sex); arrest characteristics (witness status, bystander cardiopulmonary resuscitation); locations (public or nonpublic); records on automated external defibrillator (shockable or nonshockable, chest compression fraction); out-of-hospital treatment, including airway types and medication used; patient records from the emergency medical service receiving hospitals; patient outcomes (out-of-hospital ROSC, sustained ROSC [≥2 hours], neurologic status at discharge), and time factors (response time, scene time, transport time, call to airway time [defined as the gap between the call of the dispatch center and completion of advanced airway insertion]).

### Sample Size

Initially, we estimated enrolling 852 patients to detect the expected sustained ROSC rate of 24.4% for SGA and 30.8% for ETI based on the background data in Taipei emergency medical services, to have 80% power and a 2-sided α level of 0.05. A further 10% patients were then enrolled for the potential influence of missing outcomes. We re-examined the estimated sample size of 1528 patients according to the initial parameters.

### Statistical Analysis

To compare the outcomes of the 2 types of airway devices, dichotomous and categorical variables are reported as the absolute sample size (percentages), whereas continuous variables are reported as mean (SD) for data compatible with normal distribution, and median (IQR) for data that violated normality. Continuous variables were compared using the *t* test, nonparametric analysis of variance, and Mann-Whitney test as appropriate. Categorical and nominal dependent variables were compared using the χ^2^ test or Fisher exact test. The effects of the 2 types of interventions among prespecified subgroups of age, presenting shockable rhythm, arrest in a public location, arrest witnessed, and time of call to airway were also analyzed. The median of the data was selected for the cutoff point of age and call to airway time.

In addition to intention-to-treat analysis, we conducted per-protocol analysis for efficacy evaluation in which the analysis retained patients only in adherence to their assigned airway group but not the crossover events. The per-protocol definition is similar to that in the previous study conducted by Wang et al.^6^

Univariate logistic regression was applied to estimate the odds ratios (ORs) for the primary analysis, and the 95% CIs were calculated. Multivariable logistic regression was applied for the significant variables in the univariate analyses. An additional clustered generalized estimating equation regression model with the same covariates as the multivariable model was applied to evaluate the potential dependent association at the ambulance team level. All tests were 2-sided, and a *P* value <.05 was considered statistically significant. The restricted cubic spline function was applied to depict the association between the time from call to airway accomplishment and primary and secondary outcomes. Logistic regression was applied to test the differences between the ETI and SGA groups of the restricted cubic spline. Data were processed and analyzed using SAS software, version 9.2 (SAS Institute Inc).

## Results

### Patient Characteristics

**Figure 1.**
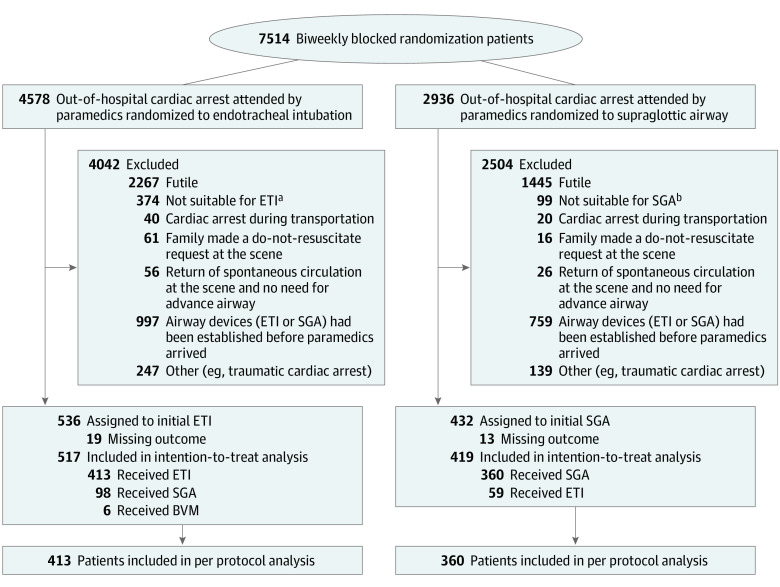
Flowchart of the Study Patients BVM indicates bag-valve-mask; ETI, endotracheal intubation; SGA, supraglottic airway. ^a^Preexisting condition, such as mouth cannot be opened wide enough for laryngoscope insertion. ^b^Preexisting condition, such as tracheostomy.

According to the intention-to-treat analysis, 7514 patients were potentially eligible and randomized to either the ETI or SGA group (Figure 1). The randomization scheme of 4 ambulance teams is shown in the eFigure in Supplement 2. Among the potentially eligible individuals, 6546 patients (87%) were excluded and 968 patients (13%) were enrolled, and the outcomes were available in 936 patients (median age, 77 [IQR, 62-85] years; 569 men [60.8%] and 367 women (39.2%]).

Reasons for exclusion were similar in both groups, except cardiac arrest during transportation en route (1.5% in the ETI group vs 0.6% in the SGA group; *P* = .001) and the airway devices (ETI or SGA) had been established before paramedics arrived (24.7% in the ETI group vs 30.3% in the SGA group; *P* < .001) (eTable 1 in Supplement 2).

The patients’ baseline characteristics are reported in Table 1. There were more patients in the ETI group (n = 517) than in the SGA group (n = 419). Mean (SD) scene time interval (18.4 [5.1] vs 16.9 [4.9] minutes) and call to airway time (15.9 [5.4] vs 13.9 [4.5] minutes) were longer for ETI than SGA. The first attempt success rates were 77% (n = 413) for ETI and 83% (n = 360) for SGA.

**Table 1.** Baseline Characteristics of the Study Groups

Demographic characteristics	Intention-to-treat, No. (%)		
	Total (n = 936)	ETI (n = 517)	SGA (n = 419)
Sex			
Male	569 (60.8)	330 (63.8)	239 (57.0)
Female	367 (39.2)	187 (36.2)	180 (43.0)
Age, y			
Mean (SD)	73.3 (28.3)	72.1 (16.4)	74.7 (38.1)
Median (IQR)	77 (62-85)	76 (62-84)	77 (63-86)
Arrest witnessed	419 (44.8)	219 (42.4)	200 (47.7)
Bystander CPR	664 (70.9)	374 (72.3)	290 (69.2)
Location			
Home	755 (80.7)	410 (79.3)	345 (82.3)
Nursing home	48 (5.1)	35 (6.8)	13 (3.1)
Public	133 (14.2)	72 (13.9)	61 (14.6)
EMS treatment			
Drug	409 (43.7)	241 (46.6)	168 (40.1)
Shockable rhythm	147 (15.7)	95 (18.4)	52 (12.4)
PAD	20 (2.1)	13 (2.5)	7 (1.7)
CCF (n = 42/51), mean (SD), %^a^	70.4 (12.9)	72.1 (13.2)	69.0 (12.6)
Time intervals, mean (SD)			
Response time	7.0 (3.4)	7.0 (3.3)	7.0 (3.5)
Scene time	17.7 (5.1)	18.4 (5.1)	16.9 (4.9)
Transport time	4.6 (3.3)	4.5 (2.3)	4.7 (4.2)
Call to airway (n = 790)^b^			
Mean (SD)	15.0 (5.1)	15.9 (5.4)	13.9 (4.5)
Median (IQR)	14 (11-18)	15 (12-19)	13 (11-16)

Abbreviations: CCF, chest compression fraction; CPR, cardiopulmonary resuscitation; EMS, emergency medical service; ETI, endotracheal intubation; PAD, public access defibrillation; SGA, supraglottic airway.

^a^ Analysis of automated external defibrillator records in the first 100 enrolled events.

^b^ Call to airway: gap between the call of dispatch center and completion of advanced airway insertion.

### Primary and Secondary Outcomes

Among the 968 enrolled patients, the primary outcome was available in 936 patients (96.7%). Missing outcomes accounted for 32 patients (19 in the ETI group, 13 in the SGA group). Sustained ROSC was 26.9% (139 of 517 patients) in the ETI group vs 25.8% (108 of 419 patients) in the SGA group. The OR of sustained ROSC was 1.02 (95% CI, 0.98-1.06) for the ETI group compared with the SGA group under the clustered generalized estimating equation model (Table 2). The OR of the secondary outcome of prehospital ROSC was 1.04 (95% CI, 1.02-1.07) for the ETI group compared with the SGA group. Other secondary outcomes, including survival to hospital discharge (OR, 1.00; 95% CI, 0.94-1.06) and good neurological outcome (cerebral performance category score ≤2) (OR, 0.99; 95% CI, 0.94-1.03), were not significantly different between the study groups. We also conducted per-protocol analysis, which revealed similar results to the intention-to-treat analysis (eTable 2 in Supplement 2).

**Table 2.** Primary and Secondary Outcomes

Outcomes	Intention-to-treat, No. (%)			OR (95% CI)	
	Total (n = 936)	ETI (n = 517)	SGA (n = 419)	Crude	Adjusted
Primary					
Sustained ROSC	247 (26.4)	139 (26.9)	108 (25.8)	1.06 (0.79-1.42)	1.02 (0.98-1.06)
Secondary					
Prehospital ROSC	82 (8.8)	55 (10.6)	27 (6.4)	1.73 (1.07-2.79)^a^	1.04 (1.02-1.07)^a^
Survival to discharge	79 (8.4)	44 (8.5)	35 (8.4)	1.03 (0.65-1.64)	1.00 (0.94-1.06)
CPC score ≤2	40 (4.3)	20 (3.9)	20 (4.8)	0.81 (0.43-1.52)	0.99 (0.94-1.03)

Abbreviations: CPC, cerebral performance category; ETI, endotracheal intubation; OR, odds ratio; ROSC, return of spontaneous circulation; SGA, supraglottic airway.

^a^ *P* < .05.

### Subgroup Analysis

In patients who received ETI, the primary outcome of sustained ROSC was not statistically significant. Although some prespecified subgroups were associated with an increased likelihood of prehospital ROSC compared with SGA (Table 3), which included patients with nonpublic arrest (OR, 2.13; 95% CI, 1.21-3.74), witnessed arrest (OR, 1.94; 95% CI, 1.12-3.34), and age 77 years or older (OR, 3.57; 95% CI, 1.53-8.34). In addition to the aforementioned subgroups, nonshockable rhythm (OR, 1.92; 95% CI, 1.05-3.51) and call to airway time less than 14 minutes (OR, 2.32; 95% CI, 1.06-5.09) were associated with prehospital ROSC in the per-protocol analysis (eTable 3 in Supplement 2). There was interaction between interventions and the subgroup of age (*P* for interaction = .03 in the intention-to-treat analysis and *P* for interaction < .01 in the per-protocol analysis) (eTable 4 in Supplement 2).

**Table 3.** Subgroup Analysis of ETI Effect Among Different Subgroups

Variable	No.	Intention-to-treat ETI effect, OR (95% CI)			
		ROSC		Survival to discharge	CPC score ≤2
		Sustained	Prehospital		
Shockable rhythm					
Yes	147	1.38 (0.69-2.76)	2.51 (0.88-7.13)	1.28 (0.57-2.97)	0.88 (0.34-2.28)
No	789	0.93 (0.67-1.30)	1.42 (0.82-2.47)	0.76 (0.42-1.37)	0.50 (0.20-1.29)
Public location					
Yes	133	0.67 (0.33-1.34)	0.93 (0.35-2.47)	0.55 (0.23-1.31)	0.78 (0.26-2.37)
No	803	1.18 (0.85-1.64)	2.13 (1.21-3.74)^a^	1.37 (0.77-2.43)	0.81 (0.37-1.78)
Arrest witnessed					
Yes	419	1.23 (0.83-1.83)	1.94 (1.12-3.34)^a^	1.20 (0.69-2.09)	0.90 (0.43-1.90)
No	517	1.02 (0.63-1.65)	2.06 (0.65-6.56)	0.90 (0.37-2.22)	0.74 (0.21-2.59)
Call to airway time					
<14	362	1.31 (0.81-2.11)	2.03 (0.97-4.25)	1.34 (0.65-2.78)	1.31 (0.45-3.83)
≥14	454	1.03 (0.66-1.62)	1.63 (0.79-3.36)	0.86 (0.42-1.80)	0.58 (0.23-1.50)
Age, y					
<77	466	0.84 (0.57-1.24)	1.09 (0.59-2.00)	0.99 (0.57-1.72)	0.89 (0.44-1.78)
≥77	470	1.43 (0.91-2.26)	3.57 (1.53-8.34)^a^^,^^b^	1.11 (0.46-2.70)	0.41 (0.08-2.28)

Abbreviations: CPC, cerebral performance category; ETI, endotracheal intubation; OR, odds ratio; ROSC, return of spontaneous circulation.

^a^ *P* < .05.

^b^ The *P* value for interaction test is .03.

### Additional Analysis

**Figure 2.**
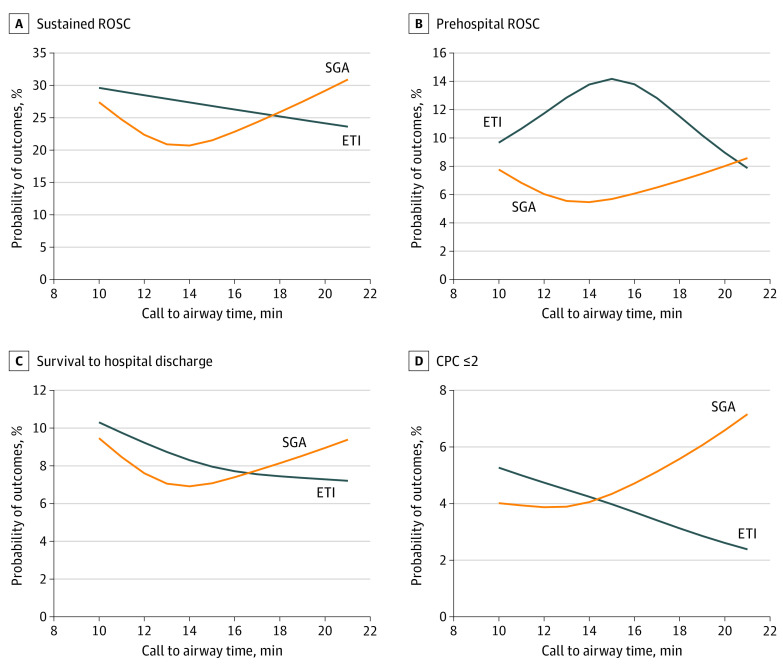
The Association Between the Probability of Outcomes and Call to Airway Time Findings were nonsignificant for sustained return of spontaneous circulation (ROSC), *P* = .44 (A); significant for prehospital ROSC, *P* = .03 (B); nonsignificant for survival to hospital discharge, *P* = .82 (C); and nonsignificant for cerebral performance category (CPC) score less than or equal to 2, *P* = .67 (D). ETI indicates endotracheal intubation; SGA, supraglottic airway.

The association between the probability of outcomes and call to airway time is also shown in restricted cubic splines in Figure 2. The probability of prehospital ROSC was higher in the ETI group than in the SGA group with statistical significance (*P* for trend = .03). Other outcomes, including sustained ROSC, survival to discharge, and good neurological outcomes (cerebral performance category score ≤2), were not significantly different between the study groups. The crossover of the effect of ETI and SGA in sustained ROSC and good neurological outcome (cerebral performance category score ≤2) may be due to the relatively small number of patients with these conditions. Furthermore, the rate of favorable outcomes tended to decline with increasing call to airway time in the ETI group but not in the SGA group.

## Discussion

In this biweekly clustered randomized clinical trial, no significant difference was found between the SGA and ETI groups for the primary outcome of sustained ROSC (ROSC≥2 hours). There was a statistically significant difference in the secondary outcome of prehospital ROSC that favored the ETI group.

Compared with individuals from Western countries, the initial shockable rhythm prevalence in Asian patients with OHCA is relatively low.^8,12^ Initial shockable rhythm in our study accounted for only 15.7% of OHCA, in contrast to 19% and 23% in 2 randomized clinical trials in the US and England.^5,6^ Compared with cardiac-cause cardiac arrest in which defibrillation alone may be sufficient,^13,14^ noncardiac causes of arrest may benefit from other prehospital management, including ETI. Per-protocol analysis of the subgroups also supported this hypothesis that ETI insertion favored prehospital ROSC in patients with nonshockable rhythm. Further research is warranted to clarify the effect of airway management strategies and different causes of cardiac arrest.

Although ETI is a complex procedure^15^ that requires experienced operators to perform, the Taipei City paramedics were the earliest trained and authorized paramedics to perform out-of-hospital intubation among metropolitan areas in Asia. In addition, protocol-based single-attempt intubation and end-tidal capnographic data to prevent esophageal intubation were applied during advanced airway insertion. An annual airway retraining program is also required for quality control. The high success rate of ETI (77%) in our study could be attributed to the aforementioned methods, compared with the randomized clinical trial conducted by Wang et al^6^ that the first-attempt success rate was low at 51%. Whether the result noted by Wang et al was influenced by the low first attempt rate, it was clear that an unsuccessful initial intubation attempt was associated with worse outcomes.^16^ In addition, the influence of advanced airway insertion on the chest compression fraction was difficult to evaluate owing to the lack of data in the Wang et al^6^ trial. Despite few cardiopulmonary resuscitation process data collected in our trial (the first 100 enrolled events, via automated external defibrillator), there was no significant difference between the chest compression fraction study groups.

Endotracheal intubation is associated with prolonged scene time intervals and time for airway insertion, according to previous reports.^6,17^ Scene time interval and call to airway time were longer in the ETI group than the SGA group in our trial. Prolonged scene time interval and time for airway insertion are associated with worse outcomes^18,19^ and the effect of ETI may be diluted in these circumstances. To clarify whether the alignment of call to airway time between the ETI and SGA groups would change the outcomes, an additional analysis was performed, as shown in restricted cubic splines. The probability of prehospital ROSC in the ETI group was higher than that in the SGA group. It is unclear whether a stepwise and algorithmic ETI training program could reduce the time in the field and the time for advanced airway insertion, and further research is warranted.

Despite the potential benefit of ETI in prehospital ROSC, generalizability should be taken into consideration owing to the type of SGA (ie, i-gel) in our trial and the paramedic-based emergency medical service systems (ie, not physician-led) may differ from other countries. Nevertheless, the principle of advanced airway insertion and the essence of prehospital management may not differ significantly between countries.

### Limitations

This study has limitations. First, the number of patients was different in the 2 groups, probably owing to the 3:2 ratios during allocation, and fewer than expected patients in the ETI group were excluded. The 3:2 ratios were based on the baseline success rate between the ETI and SGA groups in a previous report of a study in Taipei City (60% vs 90%).^8^ The fewer than expected patients excluded in the ETI group may also indicate that the intubation success rate has increased in recent years. Second, despite similar basic life support and airway management techniques in the field, in-hospital management may differ between hospitals and may potentially influence the results. Third, the study was underpowered to detect the true difference of primary outcome owing to inaccurate sample size estimation at the early stage of the design. However, even if we had realized that the sample size was inadequate at that time, we would not have been able to recruit more cases because of the outbreak of COVID-19. A total of 968 patients were included in the final analysis; thus, it is the largest sample size we could have achieved under the circumstances.

## Conclusions

In this randomized clinical trial, among patients with OHCA, the initial airway management with ETI did not result in a favorable outcome of sustained ROSC compared with insertion of an SGA device. However, ETI was associated with a higher probability of prehospital ROSC compared with SGA, especially among the subgroups of nonshockable rhythm, nonpublic collapse, arrested witnessed, call to airway time less than 14 minutes, and age 77 years or older.

## Notes

Supplement 1.
Trial Protocol

Supplement 2. **eTable 1.** Reasons for Exclusion in the Primary Analysis **eTable 2.** The Odds Ratios of Primary and Secondary Outcomes (Per-Protocol Analysis) **eTable 3.** Subgroup Analysis of Endotracheal Intubation Effect Among Different Subgroups (Per-Protocol Analysis) **eTable 4.** Interaction Test Between Interventions and Subgroups **eFigure.** Randomization Scheme of ALS Ambulance Teams

Supplement 3.
Data Sharing Statement
