# Supplementary material for: Effect of Placement of a Supraglottic Airway Device vs Endotracheal Intubation on Return of Spontaneous Circulation in Adults With Out-of-Hospital Cardiac Arrest in Taipei, Taiwan: A Cluster Randomized Clinical Trial
Source: JAMA Netw Open. 2022 Feb 18;5(2):e2148871. doi: 10.1001/jamanetworkopen.2021.48871 (PMC8857689; doi:10.1001/jamanetworkopen.2021.48871)
Supplement: Supplement 1. — Trial Protocol [file jamanetwopen-e2148871-s001.pdf]

# A randomized controlled trial of the clinical outcomes of Supraglottic Airway device Versus Endotracheal intubation in the prehospital airway management of patients with out-of-hospital cardiac arrest (SAVE trial)

# Clinical Trial Protocol

## Table of Content


1. ACRONYMS AND ABBREVIATIONS ---------------------------------------------2
2. TRIAL SUMMARY ---------------------------------------------2
3. BACKGROUND ---------------------------------------------2
4. TRIAL DESIGN ---------------------------------------------3
   - 4.1. HYPOTHESIS ---------------------------------------------3
   - 4.2. INCLUSION CRITERIA ---------------------------------------------3
   - 4.3. EXCLUSION CRITERIA ---------------------------------------------4
   - 4.4. TRIAL SETTING ---------------------------------------------4
   - 4.5. RANDOMIZATION AND BLINDING ---------------------------------------------4
   - 4.6. EXPOSURE MANAGEMENT ---------------------------------------------4
     - 4.6.1. Intervention group ---------------------------------------------4
     - 4.6.2. Control group ---------------------------------------------5
   - 4.7. OUTCOMES MEASUREMENT ---------------------------------------------5
     - 4.7.1. Primary outcome ---------------------------------------------5
     - 4.7.2. Secondary outcomes ---------------------------------------------5
5. SAMPLE SIZE ESTIMATION ---------------------------------------------5
6. INTERIM ANALYSIS AND STOPPING CRITERIA ---------------------------------------------5
7. DATA COLLECTION ---------------------------------------------5
8. TIMELINE ---------------------------------------------6
   - 8.1. YEAR 1 ---------------------------------------------6
   - 8.2. YEAR 2 ---------------------------------------------6
   - 8.3. YEAR 3 ---------------------------------------------6
9. ANALYTIC PLAN ---------------------------------------------6
   - 9.1. PRIMARY OUTCOME ---------------------------------------------6
   - 9.2. SECONDARY OUTCOMES ---------------------------------------------6
   - 9.3. SUBGROUP ANALYSIS ---------------------------------------------7
10. REFERENCE ---------------------------------------------7

## 1. Acronyms and Abbreviations

| | |
|---|---|
| ALS | Advanced life support |
| BVM | Bag valve mask |
| CPC | Cerebral performance category |
| CPR | Cardiopulmonary resuscitation |
| EMS | Emergency medical service |
| ETI | Endotracheal intubation |
| OHCA | Out of hospital cardiac arrest |
| RCT | Randomized controlled trial |
| ROSC | Return of Spontaneous Circulation |
| SGA | Supraglottic Airway |

## 2. Trial summary

Overall survival of patients after out-of-hospital cardiac arrest (OHCA) is less than 10% worldwide. Interventions provided by emergency medical system (EMS) before arrival at hospital are of paramount importance to patient outcomes after OHCA. Among those interventions, the pros-and-cons of different prehospital advanced airways, including supraglottic airway (SGA) or endotracheal tube, remained the issue of most under debate. The objective of this study is to determine the comparative effective of endotracheal intubation (ETI) vs SGA after OHCA by conducting a randomized controlled trial (RCT) in Taipei EMS.

In this 3-year successive research plan, we will conduct a prehospital randomized controlled trial to address the following question (in Population-Intervention-Comparison-Outcome style): In adult patients with non-traumatic cause of OHCA resuscitated by emergency medical technician paramedic in the prehospital setting, will receiving ETI cause a better chance of sustained recovery of spontaneous circulation and other survival outcomes like neurologically favorable status, comparing to those who receiving SGA device. To name in short, which was called "SAVE trial", standing for **S**upraglottic **A**irway **V**ersus **E**ndotracheal tube.

According to the previous literature, background data, and previous experience in conducting the very first prehospital RCT in Taiwan and Asia by our research team, we estimated the sample size as 852 patients of OHCA to detect the primary outcome difference, and will take a 34-month period to complete the enrollment. As we know, conducting RCT especially in the prehospital setting is a pain-taking, time-consuming task but it is also a must-be-done to answer the most important question in the prehospital airway management by paramedics for patients with OHCA.

Expected results from this "SAVE trial" can figure out the best strategy of prehospital advanced airway in our own and worldwide EMS with similar configuration, therefore find out the promising chance to improve outcomes of patients with OHCA.

## 3. Background

Resuscitation of out-of-hospital cardiac arrest (OHCA) patients is one of the most important issues in emergency medical service (EMS) care.

During cardiac arrest, the brain will be damaged to varying degrees due to low blood oxygen and circulation. High-quality cardiopulmonary resuscitation (CPR) is vital to

reduce the damage of cardiac arrest. Besides, effective ventilation is the basis to maintain the blood oxygenation in the brain, thereby reducing hypoxia and brain damage, and can increase the possibility of return of spontaneous circulation (ROSC) and the chance of neurologically intact survival. [1, 2]

For cardiac patients, the "chain of survival" emphasized early recognition, early CPR, early defibrillation, early advanced life support and post arrest care. There is no doubt about the benefits of early CPR and defibrillation. [3-5] While the prehospital airway management of OHCA patients remained controversial. The airway management in prehospital settings can be divided into basic and advanced techniques. The basic technique includes bag valve mask (BVM) and the advanced technique includes both supraglottic airway device (SGA) and endotracheal intubation (ETI).

The advantage of advanced airway management is to provide a definite and secure way of oxygen delivery in the first aid of sudden cardiac arrest patients. Compared to BVM, advanced airway management may further reduce the interruption during ambulance transport. Advanced care paramedics in Taiwan were authorized to perform either SGA or ETI in the prehospital setting. While ETI may have some pitfalls like esophageal intubation, CPR interruption and may further influence a patient's outcome. [6-10] Theoretically, the SGA can be established quickly, and some studies have also confirmed that the SGA is better than ETI in resuscitation and can reduce the interruption during CPR. [11] However, such inferences were questioned in the following studies. Compared with SGA, ETI achieved higher sustained ROSC (odds ratio [OR] 1.35; 95% confidence interval [CI] 1.19–1.54) according to the analysis from Cardiac Arrest Registry to Enhance Survival (CARES) registry. [12] A recent meta-analysis included 34,533 ETI patients and 41,116 SGA patients that revealed ETI had statistically significant higher odds of ROSC (OR 1.28, 95% CI 1.05-1.55) compared to SGA, while no RCT exists to compare these two airway interventions, resulting in an overall low quality of evidence. [13]

In summary, previous conclusions were the result of retrospective studies, and there are interference factors that cannot be corrected innately in the research design, such as the airway management techniques of paramedics, the quality of chest compression, different types of airway devices, EMS protocols, etc. Therefore, inconsistent conclusions of previous studies appeared and RCT was the best way to further clarify the effect of prehospital airway management between SGA and ETI.

## 4. Trial Design

This trial is a randomized controlled trial and will use cluster randomization with periodic crossover.

### 4.1. Hypothesis

The hypothesis of the trial was illustrated by the PICO style, (<u>P</u>opulation-<u>I</u>ntervention-<u>C</u>omparison-<u>O</u>utcome). The non-traumatic OHCA patients (Population) in Taipei city who receive ETI(Intervention) can achieve higher sustained ROSC (Outcome) rate comparing to patients who receive SGA insertion (Comparison).

### 4.2. Inclusion Criteria: the trial will include patients meeting all of the following conditions

- Out-of-hospital cardiac arrest
- Non-traumatic etiology
- Age ≥20 years
- Treat by participating ALS teams' paramedics
- Require advanced airway management

### 4.3. Exclusion Criteria: the trial will exclude patients meeting one of the following conditions

- Resuscitation deemed inappropriate: rigor mortis or livor mortis
- Not suitable for ETI: like facial deformity, mouth nearly open
- Not suitable for SGA: like preexisting tracheostomy, known upper airway obstruction with a foreign body, anaphylactic laryngeal edema
- Cardiac arrest during transportation en route
- Family made a do-not-resuscitate request at the scene
- ROSC at the scene and no need for advanced airway
- Paramedic participation in the study second on scene and airway management already been established

### 4.4. Trial setting

Paramedics who work in the four ALS teams in Taipei city will be enrolled automatically.

The four ALS teams are staffed by approximately 120 paramedics who are trained and authorized to perform ETI/ SGA insertion and intravenous injections of medications. Paramedics have completed 1,280 hours of training, according to the requirements of the Taiwan Ministry of Health and Welfare.

Taipei City is a metropolitan area with a population around 2.65 million, and up to 3.0 million including the inflow of daytime workers, within its 272-km2 area.
The majority of the population is Taiwanese or Chinese.

### 4.5. Randomization and blinding

Four ALS teams will be divided into two randomization clusters. Each cluster is assigned to either ETI or SGA in a biweekly period. The study center will instruct the clusters to alternatively change to either the ETI or SGA according to the sequence random allocation.

To guarantee a balanced case number in the final analysis. The allocation periods of ETI vs. SGA in the fashion of three periods of ETI to two periods of SGA (3:2 ratio), which is based on the baseline first attempt success rates of ETI and SGA in the Taipei EMS system (i.e., the success rate of first attempt: 60% of ETI vs. 90% of SGA).

The blinding of paramedics is not possible due to the airway management devices cannot be concealed.

### 4.6. Exposure management

#### 4.6.1. Intervention group

The intervention arm of the trial is primary ETI in eligible patients according to inclusion criteria. The endotracheal tube will be inserted via direct laryngoscopy but

not video laryngoscopy. There is no additional training course for paramedics who are involved in this trial.
The confirmation of success ETI is mainly by end-tidal capnographic data and auscultation. Protocol-based single-attempt intubation is also applied.
If the first advanced airway attempt fails, rescue airway management including SGA insertion or BVM ventilation are allowed.

#### 4.6.2. Control group
The control arm of the trial is primary SGA insertion in eligible patients according to inclusion criteria. The SGA which is used in the trial is second-generation supraglottic airway device from Intersurgical (i.e., i-gel). There is no additional training course for paramedics who are involved in this trial. Misplacement of SGA is less common due to its design to create an anatomical seal of the pharyngeal, laryngeal and perilaryngeal structures. Protocol-based single-attempt SGA insertion is also applied.
If the first advanced airway attempt fails, rescue airway management including ETI or BVM ventilation are allowed.

### 4.7. Outcomes measurement
#### 4.7.1. Primary outcome:
The primary outcome of the trial is sustained ROSC (defined as ROSC ≥2 h). Due to overcrowded condition in Taiwan's emergency departments, patient's admission could be delayed. Therefore, sustained ROSC was used and was well-validated in previous study in Taiwan. [14]

#### 4.7.2. Secondary outcomes:
The following secondary outcomes will be analyzed during the trial:
prehospital ROSC, survival to hospital discharge, favorable neurological outcome (defined as cerebral performance category scores 1 and 2, CPC ≤2).

## 5. Sample size estimation
The sample size estimation is based on the primary outcome of sustained ROSC. According to previous study conducted in Taipei, sustained ROSC rate was 24.4% for SGA and 30.8 % for ETI. [15]
We estimated to enroll 852 patients to detect the absolute difference of 6.4% between the SGA and ETI group to have 80% power and a two-sided α-level of 0.05. Considering the number of OHCA patients treated by the participating ALS teams in Taipei is about 300 patients annually, we expect to have adequate number of patients of the three years study period.

## 6. Interim analysis and stopping criteria
Based on the research ethics, the interim analysis will be done between 10 to 20 months after the recruitment; if there is a beneficial prognostic trend, the trial will continue, otherwise it will be terminated.
This research has entrusted Taiwan Clinical Trial Bioinformatics and Statistical Center (which is founded by National Research Program for Biopharmaceuticals at the Ministry of Science and Technology of Taiwan) to provide interim analysis and final analysis assistance.

## 7. Data collection:

The data will be collected at the Utstein-style cardiac arrest registry system in Taipei, which consisted of the following items: patient demographics (age and sex); arrest characteristics (witness status, bystander CPR), locations (public or non-public), records on automated external defibrillator (shockable or non-shockable, chest compression fraction); out-of-hospital treatment, including airway types and medication used; patient records from the EMS receiving hospitals; patient outcomes (out-of-hospital return of spontaneous circulation, sustained return of spontaneous circulation [≥2 hours], neurologic status at discharge), and time factors (response time, scene time, transport time, call to airway time [defined as the gap between the call of the dispatch center and advanced airway insertion completion]).

## 8. Timeline

### 8.1. Year 1

- Call expert meetings to achieve consensus on research
- Obtain Institutional review board approval of waiver of informed consent
- Prepare and start to allocation
- Establish relevant data collection for the trial

### 8.2. Year 2

- Call regular expert meetings to follow the progress of the trial
- Regularly report to the Institutional review board
- Regularly analyze and improve the compliance of the participating paramedics
- Conduct the first-time interim analysis

### 8.3. Year 3

- Call regular expert meetings to follow the progress of the research and disclose the final research results
- Conduct the second-time interim analysis
- Complete allocation and also start preliminary data analysis

## 9. Analytic plan

The statistical analysis methods include descriptive analysis, T-test, Chi-squared test, logistic regression, and propensity score analysis.

For the effect of airway management devices between ETI and SGA, odds ratios and 95% confidence intervals will be calculated. All tests are two-sided, and a p-value <0.05, will be considered statistically significant.

The analysis will be done according to both intention-to-treat and as-treated principles, and reported according to the CONSORT guidelines.

### 9.1. Primary outcome:

The primary outcome of sustained ROSC will be analyzed and calculated the odds ratio of ETI vs SGA. 95% confidence intervals will be used to quantify the treatment effect.

### 9.2. Secondary outcomes:

Secondary outcomes include prehospital ROSC, survival to hospital discharge, and favorable neurological outcome will also be analyzed in a similar manner.

### 9.3. Subgroup analysis

Several subgroups will be analyzed to explore the association of different airway management devices, including patient's age, presenting shockable rhythm, arrest in a public location, arrest witnessed, and time of call to airway. The median will be chosen for analyzing for the call to airway time and the age of patients.

## 10. Reference


1. Yeh, S.T., et al., *Oxygen requirement during cardiopulmonary resuscitation (CPR) to effect return of spontaneous circulation.* Resuscitation, 2009. **80**(8): p. 951-5.
2. Idris, A.H., et al., *Does hypoxia or hypercarbia independently affect resuscitation from cardiac arrest?* Chest, 1995. **108**(2): p. 522-8.
3. Ong, M.E., et al., *Pan-Asian Resuscitation Outcomes Study (PAROS): rationale, methodology, and implementation.* Acad Emerg Med, 2011. **18**(8): p. 890-7.
4. Ecc Committee, S. and A. Task Forces of the American Heart, *2005 American Heart Association Guidelines for Cardiopulmonary Resuscitation and Emergency Cardiovascular Care.* Circulation, 2005. **112**(24 Suppl): p. IV1-203.
5. Eisenberg, M.S. and T.J. Mengert, *Cardiac resuscitation.* N Engl J Med, 2001. **344**(17): p. 1304-13.
6. Dunford, J.V., et al., *Incidence of transient hypoxia and pulse rate reactivity during paramedic rapid sequence intubation.* Ann Emerg Med, 2003. **42**(6): p. 721-8.
7. Katz, S.H. and J.L. Falk, *Misplaced endotracheal tubes by paramedics in an urban emergency medical services system.* Ann Emerg Med, 2001. **37**(1): p. 32-7.
8. Wang, H.E., et al., *Outcomes after out-of-hospital endotracheal intubation errors.* Resuscitation, 2009. **80**(1): p. 50-5.
9. Wang, H.E., et al., *Preliminary experience with a prospective, multi-centered evaluation of out-of-hospital endotracheal intubation.* Resuscitation, 2003. **58**(1): p. 49-58.
10. Wang, H.E., et al., *Paramedic intubation errors: isolated events or symptoms of larger problems?* Health Aff (Millwood), 2006. **25**(2): p. 501-9.
11. Kurola, J., et al., *Airway management in cardiac arrest--comparison of the laryngeal tube, tracheal intubation and bag-valve mask ventilation in emergency medical training.* Resuscitation, 2004. **61**(2): p. 149-53.
12. McMullan, J., et al., *Airway management and out-of-hospital cardiac arrest outcome in the CARES registry.* Resuscitation, 2014. **85**(5): p. 617-22.
13. Benoit, J.L., et al., *Endotracheal intubation versus supraglottic airway placement in out-of-hospital cardiac arrest: A meta-analysis.* Resuscitation, 2015. **93**: p. 20-6.
14. Ma, M.H., et al., *A randomized trial of compression first or analyze first strategies in patients with out-of-hospital cardiac arrest: results from an Asian community.* Resuscitation, 2012. **83**(7): p. 806-12.
15. Ministry of Science and Technology, R.O.C., *Research on the best approach for airway management in patients with out-of-hospital cardiac arrest.* 2015.
