# Supplementary material for: Effect of Placement of a Supraglottic Airway Device vs Endotracheal Intubation on Return of Spontaneous Circulation in Adults With Out-of-Hospital Cardiac Arrest in Taipei, Taiwan: A Cluster Randomized Clinical Trial
Source: JAMA Netw Open. 2022 Feb 18;5(2):e2148871. doi: 10.1001/jamanetworkopen.2021.48871 (PMC8857689; doi:10.1001/jamanetworkopen.2021.48871)
Supplement: Supplement 2. — eTable 1. Reasons for Exclusion in the Primary Analysis eTable 2. The Odds Ratios of Primary and Secondary Outcomes (Per-Protocol Analysis) eTable 3. Subgroup Analysis of Endotracheal Intubation Effect Among Different Subgroups (Per-Protocol Analysis) eTable 4. Interaction Test Between Interventions and Subgroups eFigure. Randomization Scheme of ALS Ambulance Teams [file jamanetwopen-e2148871-s002.pdf]

Supplementary Online Content

Lee A-F, Chien Y-C, Lee B-C, et al. Effect of placement of a supraglottic airway device vs endotracheal intubation on return of spontaneous circulation in adults with out-of-hospital cardiac arrest in Taipei, Taiwan: a cluster randomized clinical trial. *JAMA Netw Open*. 2022;5(2):e2148871. doi:10.1001/jamanetworkopen.2021.48871

eTable 1. Reasons for Exclusion in the Primary Analysis
eTable 2. The Odds Ratios of Primary and Secondary Outcomes (Per-Protocol Analysis)
eTable 3. Subgroup Analysis of Endotracheal Intubation Effect Among Different Subgroups (Per-Protocol Analysis)
eTable 4. Interaction Test Between Interventions and Subgroups
eFigure. Randomization Scheme of ALS Ambulance Teams

This supplementary material has been provided by the authors to give readers additional information about their work.

© 2022 Lee A-F et al. *JAMA Network Open.*

eTable1. Reasons for exclusion in the primary analysis

(n, %)	Total (n=6546)	ETI (n=4042)	SGA (n=2504)	p-value
Futile	3,712 (56.7%)	2,267 (56.1%)	1,445 (57.7%)	0.200
Not suitable for ETI (inability to open their mouth wide enough for laryngoscope insertion)	374 (5.7%)	374 (9.3%)	0 (0.0%)	-
Not suitable for SGA (preexisting tracheostomy)	99 (1.5%)	0 (0.0%)	99 (4.0%)	-
Cardiac arrest during transportation en route	77 (1.2%)	61 (1.5%)	16 (0.6%)	0.001*
Family made a do-not-resuscitate request at the scene	60 (0.9%)	40 (1.0%)	20 (0.8%)	0.505
Return of spontaneous circulation at the scene and no need for advance airway	82 (1.3%)	56 (1.4%)	26 (1.0%)	0.253
The airway devices (ETI or SGA) had been established before paramedics arrived.	1,756 (26.8%)	997 (24.7%)	759 (30.3%)	<0.001*
Others (like traumatic cardiac arrest)	386 (5.9%)	247 (6.1%)	139 (5.6%)	0.359

Abbreviations: ETI, endotracheal intubation; SGA, supraglottic airway

© 2022 Lee A-F et al. *JAMA Network Open.*

eTable 2. The odds ratios of primary and secondary outcomes (per-protocol analysis)

Outcomes	Per-protocol			Odds ratio (95%CI)	
	Total (n=773)	ETI (n=413)	SGA (n=360)	Crude OR	Adjusted OR
**Primary outcome**					
Sustained ROSC	203 (26.3%)	113 (27.4%)	90 (25.0%)	1.13 (0.82-1.56)	1.02 (0.98-1.07)
**Secondary outcomes**					
Prehospital ROSC	68 (8.8%)	47 (11.4%)	21 (5.8%)	2.07 (1.21-3.54)*	1.06 (1.03-1.09)*
Survival to discharge	60 (7.8%)	34 (8.2%)	26 (7.2%)	1.17 (0.69-1.99)	1.01 (0.97-1.07)
CPC≤2	29 (3.8%)	15 (3.6%)	14 (3.9%)	0.94 (0.45-1.98)	1.00 (0.97-1.03)

*$p < 0.05$

Abbreviations: CPC, cerebral performance category; CI, confidence interval; ETI, endotracheal intubation; OR, odds ratio; ROSC, return of spontaneous circulation; SGA, supraglottic airway

© 2022 Lee A-F et al. *JAMA Network Open.*

eTable 3. Subgroup analysis of endotracheal intubation effect among different subgroups (per-protocol analysis)

Per-protocol ETI effect Odds ratio (95% CI)		n	Sustained ROSC	Prehospital ROSC	Survival to discharge	CPC≤2
Shockable rhythm	Yes	115	1.47 (0.67-3.25)	2.16 (0.66-7.05)	1.64 (0.59-4.58)	1.43 (0.42-4.92)
	no	658	1.00 (0.70-1.44)	1.92 (1.05-3.51)*	0.85 (0.44-1.65)	0.47 (0.16-1.38)
Public location	Yes	100	0.66 (0.29-1.47)	0.86 (0.29-2.57)	0.78 (0.29-2.09)	0.93 (0.28-3.12)
	no	673	1.28 (0.90-1.83)	2.83 (1.48-5.39)*	1.49 (0.77-2.87)	0.98 (0.38-2.58)
Arrest Witnessed	Yes	345	1.32 (0.86-2.04)	2.20 (1.19-14.05)*	1.41 (0.73-2.74)	1.05 (0.42-2.65)
	no	428	1.04 (0.62-1.75)	2.79 (0.76-10.28)	0.92 (0.37-2.31)	0.83 (0.24-2.91)
Call to airway time	＜14	329	1.37 (0.83-2.23)	2.32 (1.06-5.09)*	1.47 (0.69-3.16)	1.33 (0.42-4.22)
	≧14	392	1.12 (0.69-1.81)	1.77 (0.78-4.05)	1.05 (0.46-2.42)	0.73 (0.25-2.14)
Age	＜77	381	0.87 (0.56-1.34)	0.92 (0.47-1.81)	1.14 (0.60-2.16)	0.94 (0.42-2.13)
	≧77	392	1.58 (0.97-2.59)	9.18 (2.74-30.72)*[b]	1.28 (0.48-3.42)	0.89 (0.12-6.36)

*$p < 0.05$

Abbreviations: CPC, cerebral performance category; CI, confidence interval; ETI, endotracheal intubation; ROSC, return of spontaneous circulation

b: The *P* value for interaction test is < 0.01

© 2022 Lee A-F et al. *JAMA Network Open.*

eTable 4. Interaction test between interventions and subgroups

Intention-to-treat	Sustained ROSC	Prehospital ROSC	Survival to discharge	CPC≤2
Shockable rhythm	0.32	0.35	0.32	0.42
Public location	0.14	0.15	0.09	0.96
Arrest Witnessed	0.55	0.92	0.60	0.79
Call to airway time	0.48	0.68	0.40	0.27
Age	0.08	0.03*	0.82	0.42
**Per-protocol**	**Sustained ROSC**	**Prehospital ROSC**	**Survival to discharge**	**CPC≤2**
Shockable rhythm	0.86	0.39	0.30	0.18
Public location	0.14	0.07	0.29	0.95
Arrest Witnessed	0.48	0.75	0.46	0.77
Call to airway time	0.57	0.64	0.56	0.46
Age	0.07	<0.01*	0.85	0.96

*$P < 0.05$

Abbreviations: CPC, cerebral performance category; ROSC, return of spontaneous circulation

© 2022 Lee A-F et al. *JAMA Network Open.*

eFigure 1. Randomization Scheme of ALS ambulance teams

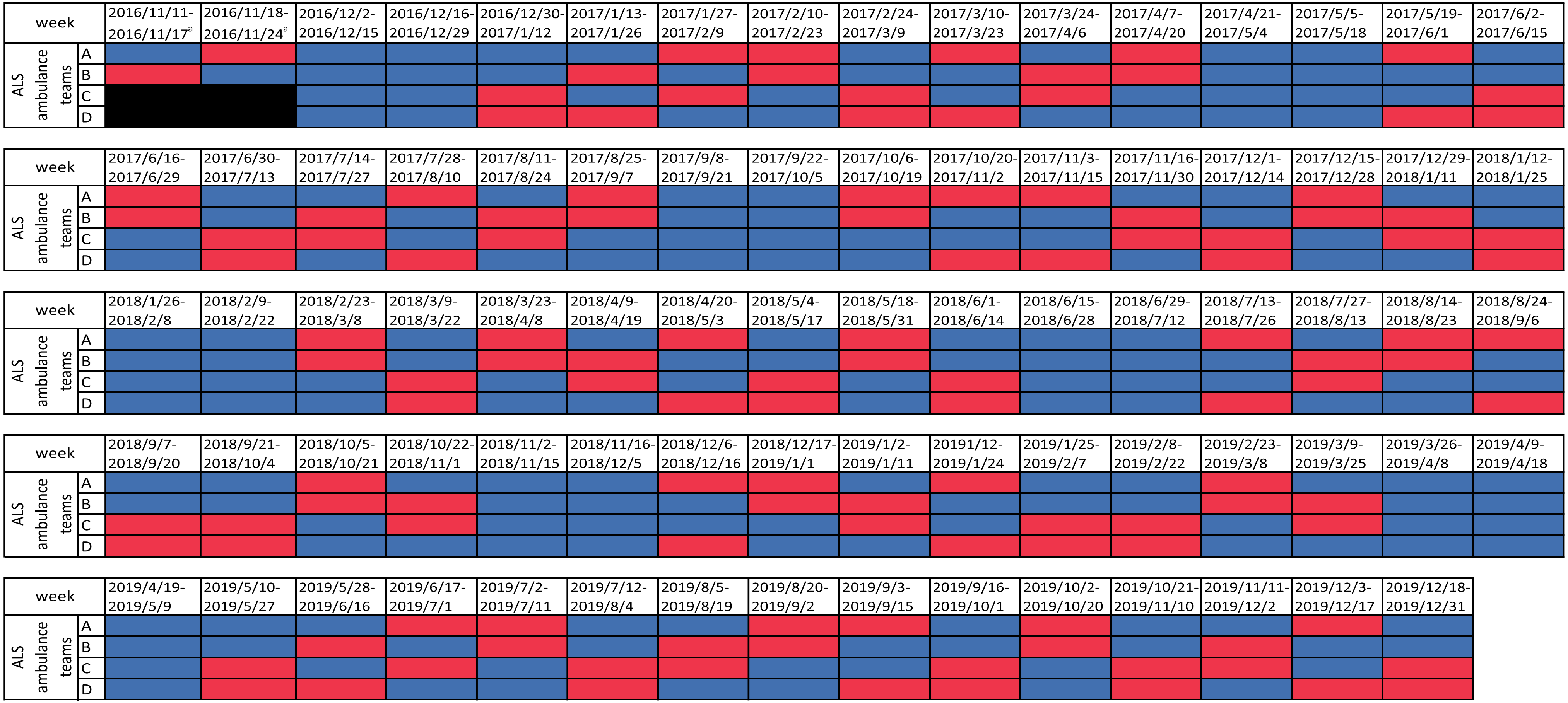

Abbreviation: ALS, advance life support

ETI

SGA

a: Preparation weeks from 2016/11/11-11/24, with only A and B ambulance teams began the tryouts

© 2022 Lee A-F et al. *JAMA Network Open.*
