# Supplementary material for: Effect of Placement of a Supraglottic Airway Device vs Endotracheal Intubation on Return of Spontaneous Circulation in Adults With Out-of-Hospital Cardiac Arrest in Taipei, Taiwan: A Cluster Randomized Clinical Trial
Source: JAMA Netw Open. 2022 Feb 18;5(2):e2148871. doi: 10.1001/jamanetworkopen.2021.48871 (PMC8857689; doi:10.1001/jamanetworkopen.2021.48871)
Supplement: Supplement 3. — Data Sharing Statement [file jamanetwopen-e2148871-s003.pdf]

Data Sharing Statement

Lee. Effect of Placement of a Supraglottic Airway Device vs Endotracheal Intubation on Return of Spontaneous Circulation in Adults With Out-of-Hospital Cardiac Arrest in Taipei, Taiwan. *JAMA Netw Open*. Published February 18, 2022. doi:10.1001/jamanetworkopen.2021.48871

Data
Data available: Yes
Data types: Deidentified participant data
How to access data: Deidentified participant data can be accessed through the contact with the corresponding authors.
When available: With publication

Supporting Documents
Document types: None

Additional Information
Who can access the data: researchers whose proposed use of the data has been approved
Types of analyses: for research purposes with permission
Mechanisms of data availability: with investigator support
